# Therapeatic evaluation and single cell analysis of adipose stromal vascular fraction isolation from a commercial cell separation system

**DOI:** 10.1186/s13287-025-04732-5

**Published:** 2025-11-07

**Authors:** Shunxin Han, Qian Zhang, Feng Lu, Junrong Cai

**Affiliations:** Department of Plastic and Cosmetic Surgery, Nanfang Hospital, Southern Medical University, 1838 Guangzhou North Road, Guangzhou, 510515 Guangdong People’s Republic of China

**Keywords:** Stromal vascular fraction, Adipose-derived stem cells, Enzyme digestion method, Separation equipment, Mechanical method, Single-nucleus RNA sequencing

## Abstract

**Background:**

In the field of regenerative therapy, the stromal vascular fraction (SVF) extracted from adipose tissue has been widely recognized for its significant benefits. However, the cellular composition and therapeutic effect of SVF products prepared via different methods are unclear.

**Methods:**

SVF cells were obtained via three approaches: (1) generation of the SVF via mechanical emulsification (M-SVF), (2) generation of the SVF via laboratory enzymatic digestion (L-SVF), and (3) generation of the SVF via commercial cell separation systems (C-SVF). We evaluated their healing effects on mouse wounds. Additionally, we utilized single-nucleus RNA sequencing (snRNA-seq) technology to explore the cellular composition of the C-SVF.

**Results:**

The cell yield of C-SVF was comparable to that of L-SVF. During in vitro culture, C-SVF exhibited enhanced proliferation and a reduced proportion of apoptotic cells. In a mouse wound model, the application of C-SVF facilitated the closure of mouse wounds and improved collagen remodeling and angiogenesis in the wound area. Additional snRNA-seq analysis revealed that APOE + adipose-derived stem cells and immune cells, especially M2 anti-inflammatory macrophages, are enriched in C-SVF, which together promote wound repair, and that APOE + adipose-derived stem cells (ADSCs) and immune cells, especially M2 anti-inflammatory macrophages, are enriched in C-SVF, which jointly regulate and promote wound repair.

**Conclusion:**

A commercial extraction system is an effective method for isolating viable SVF cells enriched with APOE + ADSCs and M2 macrophages.

## Introduction

Stem cell therapy has recently gained widespread acceptance in regenerative medicine, covering a range of treatments for conditions such as stubborn wounds, ischemic conditions, and tissue deficiencies. Adipose-derived stem cells (ADSCs) have increased in prominence as preferred stem cells because of their facile procurement, high storage capacity, and rapid proliferation kinetics [1]. The stromal vascular fraction (SVF), a recently emerged derivative of adipose tissue, has sparked widespread research endeavors. Its abundant bioactive substances, including notably ADSCs and matrix components, have positioned it as a viable solution for treating stubborn wounds [2–4].

At present, there is no broad consensus on the standard separation procedure for SVFs. Typically, separation methods can be classified into two main categories on the basis of whether collagenase is used to assist in the breakdown of the extracellular matrix (ECM) of adipocytes: enzymatic methods and nonenzymatic methods. Nonenzymatic (mechanical) methods rely mainly on physical operations such as emulsification, centrifugation, oscillation, and vortexing to disrupt the ECM and concentrate cellular components [5, 6]. The enzymatic method results in a high yield of SVF cells but is more expensive, has a longer preparation time, and has a greater risk of contamination. In contrast, nonenzymatic methods are less labor intensive, time saving, and easier to use in clinical practice; however, it is still uncertain whether they have regenerative effects comparable to those of enzymatic methods [7].

To obtain SVFs more conveniently and quickly during surgery, a range of semi or fully automatic separation and extraction devices have been introduced to the market [8]. Among these devives, Celution 800 was proven to be more effective with rapid processing time, greater viable cell yield, a lower residual enzyme level and a reduced cost [9]. Celution 800 has been widely used in different indications and has achieved significant results [10–12].

In this study, we compared the regenerative effects of SVF cells obtained via one nonenzymatic and two enzymatic methods. We evaluated their regenerative effects in a mouse wound model. Additionally, we utilized single-nucleus RNA sequencing (snRNA-seq) to explore the cellular composition of the SVF generated by commercial cell separation systems.

## Materials and methods

### Adipose tissue acquisition and processing

With the informed consent and approval of the Medical Research Ethics Committee of Nanfang Hospital, affiliated with Southern Medical University, adipose aspirates were obtained from six young healthy female human donors (demographic details of the participants are presented in Additional file 1). Following collection, the samples were immediately cooled on ice and subjected to further processing.

Preparation of the SVF generated via laboratory enzymatic digestion (L-SVF): The adipose aspirate was subjected to digestion with a 0.1% collagenase I solution (Solarbio, Beijing) in a 37 °C shaker for 30 min. The mixture was subsequently centrifuged at 1000 rpm for 5 min to isolate the cell pellet at the bottom. Following filtration through a 100 μm mesh sieve, the cells were resuspended in phosphate-buffered saline (PBS) (Servicebio, Hubei). To remove red blood cells, the samples were treated with red blood cell lysis buffer (GenStar, Beijing) at room temperature for 5 min. After another centrifugation at 1000 rpm for 5 min, the pellet was resuspended to yield the L-SVF suspension.

Preparation of the SVF generated by mechanical emulsification (M-SVF): The adipose aspirate was subjected to centrifugation at 3000 rpm for 3 min. The supernatant and top oil layer were discarded, and the intermediate layer was transferred to a threaded syringe with a 1.4 mm Luer connector. It was then injected and expelled 50 times, followed by centrifugation (3000 rpm, 3 min) to obtain M-SVF.

Preparation of SVFs generated by a commercial cell separation system (C-SVF): Approximately 250 ml of adipose aspirate was added to the Celulation 800 system, after which the Celase enzyme reagent was added. Following standardized washing and centrifugation steps, C-SVF was obtained.

### Cell morphology

The freshly obtained L-SVF and C-SVF were cultured in Dulbecco’s modified Eagle’s medium (DMEM) (Gibco, Waltham, MA) supplemented with 10% fetal bovine serum and 100 U/ml penicillin‒streptomycin. The morphology of the cells was subsequently examined via an inverted microscope.

### Cell proliferation rate

The manufacturer’s manual for assessing cell viability was followed with the Human Cholecystokinin/Octapeptide (CCK8) ELISA Kit from Guangzhou Orida Biotechnology Co., Ltd. The optical density (OD) at 450 nm is indicative of the cell proliferation potential.

### Collagenase residue detection

The manufacturer’s manual was followed to employ a Collagenase Type I Residue Detection ELISA Kit (Shanghai Ruifan Biological Technology Co., Ltd.) to identify collagenase residues. The OD reading at 450/630 nm was used to determine the concentration of the collagenase residue.

### Flow cytometry

The SVF cell suspension was prepared at a concentration of 1 × 10^6/ml, 100 µl of the suspension was removed, and the mixture was incubated with the following antibodies according to the manufacturer’s protocol: anti-CD90-APC, anti-CD105-PerCP-Cy5.5, anti-CD31-FITC, anti-CD133-PE, anti-CD146-FITC, and anti-CD34-PE-Cy7, and anti-PDGFRα-PE, Annexin V, and PI (BD BIOSCIENCE, USA; 1:200). The samples were subsequently analyzed via a flow cytometer (BD FACS Cantoll flow cytometer, USA).

### Animals

All animal experiments were approved by the Experimental Animal Care and Use Committee of Nanfang Hospital and were conducted in strict accordance with the guidelines set by the National Health and Medical Research Council (China). Female nude mice (BALB/c-nu), aged 6–8 weeks and weighing 20–23 g, were purchased from the Experimental Animal Center of Southern Medical University (Guangzhou, China). The mice were bred through a regular breeding program at the Experimental Animal Center of Southern Medical University. The animals were maintained on a standard food diet with free access to food under a 12-h light‒dark cycle.

### Establishment of a full-thickness skin defect wound model

After the mice were anesthetized with an isoflurane anesthesia machine (Yuyan Corporation, China) at a flow rate of 1 L/min, a circular full-thickness wound (diameter of 6 mm) was created through the skin on the on both sides of the dorsum of each mouse. The mice were randomly divided into four groups, namely the L-SVF, M-SVF, C-SVF, and PBS groups, with six mice in each group (*n* = 6). The experimental groups were subcutaneously injected with 0.1 ml of the corresponding SVF cell suspension (2 × 10^5 cells/ml), whereas the control group received 0.1 ml of PBS. The wound was then wrapped with sterile Tegaderm dressings (3 M Healthcare, St Paul, MN, USA), which were changed every other day until day 14. Digital photos were taken on days 0, 2, 4, 7, 10, and 14, and skin tissue around the wound was collected on days 7 and 14. The wound area was quantified via ImageJ. For the anesthetized mice from which samples have already been taken, trained and skilled personnel euthanized them via the method of cervical dislocation. Death was confirmed by absence of corneal reflex and cessation of heartbeat for 5 min. All procedures complied with ARRIVE Guidelines 2.0, including randomization/blinding/sample size protocols detailed in Supplementary The ARRIVE Essential 10, and were approved by the Experimental Animal Care and Use Committee of Nanfang Hospital (Approval No: NFEC-2024-296).

### Histological staining

The harvested tissue samples were fixed in 10% formalin for 36–48 h, and paraffin-embedded tissue sections with a thickness of 4 micrometres were prepared. Following deparaffinization, the sections were stained with hematoxylin and eosin (H&E) and Masson’s trichrome (BASMEDTSCI, Hubei) in accordance with the standard protocol and the manufacturer’s instructions. For immunohistochemical staining, antigen retrieval was conducted using an EDTA solution (pH = 9.0), followed by washing with PBS and blocking with goat serum for 1 h at room temperature to prevent nonspecific binding. The sections were subsequently incubated overnight at 4 °C with a primary antibody against CD31 (EPR17259; 1:1000; Abcam, Cambridge, MA). The next day, the sections were incubated with a secondary antibody conjugated with HRP, counterstained with hematoxylin, and developed with diaminobenzidine. Collagen content and vessel density were quantified via ImageJ software.

### Quantitative reverse transcription polymerase chain reaction (qRT-PCR)

The adipose tissue and C-SVF was homogenized in Trizol, and total RNA was isolated using the chloroform-isopropanol precipitation method following the standard protocol for Trizol-based extraction [13]. cDNA was generated by reverse transcription using the Reverse Transcription kit (Biosharp), and quantitative PCR analyses were performed in triplicate using an Q6N26785 with SYBR Green PCR master mix (Biosharp). All primer sequences or primerprobe combinations are listed in Supplementary File. The mean cycle threshold (Ct) for each gene was normalized to levels of PPIA in the same sample (dCt). Unpaired two-sample t-tests were used to determine differences in mean delta Ct values between treatment groups. The fold change was calculated by the delta-delta Ct method (fold = 2ddCt).

### snRNA-seq

The improved nuclear separation method was utilized to isolate nuclei from frozen human white adipose tissue (WAT) and C-SVF. Sequencing was conducted by SequMed Biotech, Inc. The single-cell nuclear suspension was assessed with a Countess II instrument prior to loading onto the machine, with an anticipated capture of 10,000 nuclei. Single-cell 3’ v2 chemistry was employed to produce single-cell barcoded droplets (GEMs), and upon capturing the nuclei, the GEM solution appeared as a uniform milky white liquid. The GEM solution was subsequently withdrawn and transferred to PCR tubes for reverse transcription and library construction for sequencing. The “gene expression library” was quantified via a Qubit instrument, and the fragment size of the “gene expression library” was analyzed via Qpcr. Subsequently, Illumina NovaSeq was utilized with a PE150 sequencing strategy for paired-end sequencing, with both reads being 150 bp in length. The raw image files obtained from high-throughput sequencing were converted into sequencing reads (sequenced reads) via base calling with CASAVA and stored in FASTQ format for further analysis.

#### snRNA-seq data analysis

The default parameters of Cell Ranger single-cell software (10x Genomics) were used for data alignment, unique molecular identifier (UMI) counting, cell counting and clustering analysis. The quality of the sample-specific FASTQ files was assessed via Cell Ranger’s counts. The expression level of each transcript is determined by the quantity of UMI assigned to it. The filtered gene expression matrix was then employed for downstream analysis. RStudio (v.4.4.1) was used to visualize clustering and gene expression with the Seurat software package (v.5.1.0). The uniform manifold approximation and projection (UMAP) method in Seurat software was used for dimensionality reduction. The differential gene expression among clusters was analyzed via the Seurat function FindMarkers and the Wilcoxon test. Violin plots, heatmaps and individual UMAP maps of the given genes were generated via the VlnPlot, DoHeatmap and FeaturePlot functions of the Seurat toolkit, respectively.

#### Cell type identification

Using the Seurat R package, after the initial Cell Ranger metric check, cells with < 200 genes or > 20% mitochondrial genes and single-sample data with nCount_RNA < 1,000 were excluded. After quality control, 7,691 C-SVF and 5,657 adipose tissue cells remained; 24,982 of the integrated data were retained for bioinformatics. PCA of the top 2,000 var. gene-aligned samples. In adipose tissue, C-SVF, and the integrated data, total-cell clustering was conducted at resolutions of 0.5, 0.5, and 0.2, respectively, via the “FindClusters” function. Dimensionality reduction was achieved via the RunUMAP function, and visualization was performed via UMAP. For subpopulation cell clustering, different cell types were extracted separately and clustered on the basis of their respective top 10 principal components. For adipose stem cells, the resolution was 0.5. Lymphocytes and macrophages were clustered according to their top 5 and top 6 principal components, respectively, with resolutions of 0.2 and 0.5. The marker genes for each cluster were identified via the “FindAllMarkers” function with the Wilcoxon rank-sum test. Only genes with |avg_log2FC| >0.25 and p_val < 0.05 were regarded as marker genes. Additional file 2 displays the marker genes for each cluster.

#### Differentially expressed gene (DEG) identification and gene ontology(GO) analysis

DEGs among different cell types or between C-SVF and adipose tissue for each cell type were identified using both FindMarkers and FindAllMarkers functions in Seurat, with the nonparametric two-sided Wilcoxon rank-sum test specified for statistical comparison. For multiple testing correction, p-values were adjusted using the Bonferroni-Holm method. DEGs were defined as those meeting the criteria of |avg_log2FC| >0.5 and adjusted p-value < 0.05. All identified DEGs, along with their corresponding log2 fold change (log2FC) and adjusted p-values, are detailed in Additional file 3.

GO enrichment analysis of the DEGs was performed using the enrichGO() function from the clusterProfiler package (v.4.14.4), with p-values adjusted for multiple testing via the Benjamini-Hochberg method. The results were visualized using the ggplot2 R package (v.3.5.1). Representative terms were selected from the top 20 GO terms or pathways based on adjusted p-value < 0.01. Complete GO enrichment results, including all terms with their adjusted p-values, are provided in Additional file 4.

### Statistical analyses

All the data are expressed as the means ± standard errors of the means. Statistical significance was determined via an unpaired t test for comparisons between two groups and intergroup comparisons employed two-way ANOVA with Tukey’s post-hoc test. Data are presented as mean ± SD with 95% confidence intervals for differences, analyzed using GraphPad Prism 10.0. P values < 0.05 denoted statistical significance.

## Results

### C-SVF maintained the viability of ADSCs

**Fig. 1:**
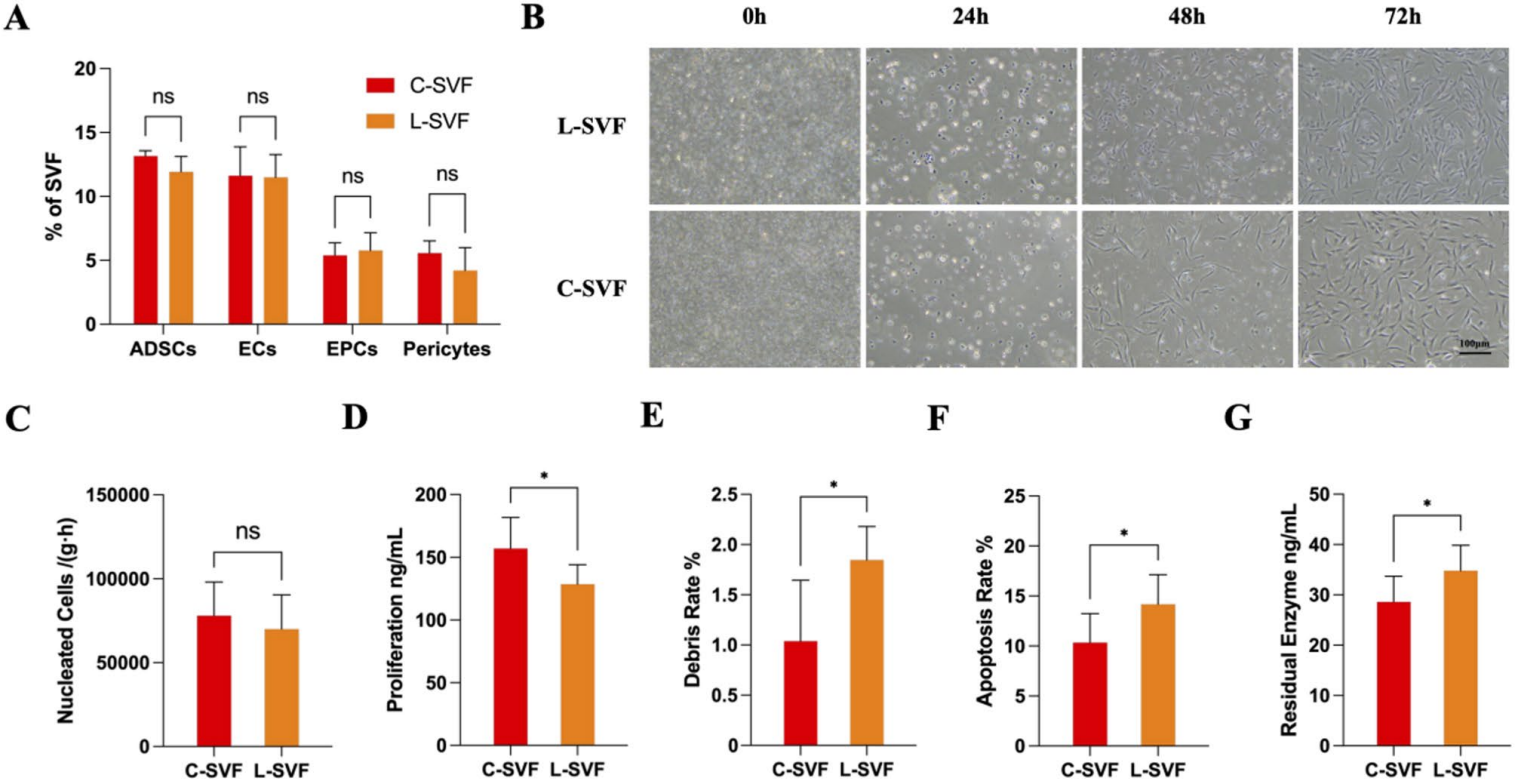
Cytological comparison between the L-SVF and C-SVF. A) Flow cytometric analysis of the principal constituents of freshly isolated cells in the L-SVF and C-SVF groups (*n* = 3). B)Morphological characteristics of P0 ADSCs generated from L-SVFs and C-SVFs. Scale bar = 100 μm. C)Viable nucleated cell yield per milliliter of processed tissue. D)Comparative analysis of cell proliferation rates between the L-SVF and C-SVF groups (*n* = 6). E)Comparative evaluation of the cell debris rates between the L-SVF and C-SVF groups (*n* = 6). F)Comparative assessment of cell apoptosis rates between the L-SVF and C-SVF groups (*n* = 6). G)Residual levels of human type I collagenase in the L-SVF and C-SVF groups (*n* = 6). (ns *p* > 0.05, *p < 0.05, **p < 0.01, ***p < 0.001)

The primary cell types in the SVF cell suspension encompass a variety of cell populations, such as ADSCs, endothelial cells (ECs), endothelial progenitor cells (EPCs), and pericytes [14]. No significant difference was found in the proportion of cellular components between L-SVF and C-SVF, suggesting that device processing does not affect the composition of different cell populations. Among each group, ADSCs constituted the major cellular population in the SVF, with the proportions of ADSCs in the L-SVF and C-SVF being 11.93 ± 1.20% and 13.16 ± 0.42%, respectively (Fig. 1A). No notable difference in the morphology of ADSCs extracted via the two methods was detected (Fig. 1B). Furthermore, the data indicate that the labotort enzymatic method yields approximately 175,000 ± 51,068 nucleated cells per gram of adipose tissue in approximately 2.5 h, whereas the Celution 800 method produces approximately 112,000 ± 35,452 nucleated cells in approximately 1.5 h. There was no statistically significant difference in extraction efficiency between the two methods (L-SVF and C-SVF produced approximately 70,000 ± 16,679 and 78,024 ± 16,342 nucleated cells per gram of adipose tissue per hour, respectively, *P* >0.05) (Fig. 1C). Notably, the proliferative capacity of C-SVF was marginally greater than that of L-SVF (*P* < 0.05) (Fig. 1D). The L-SVF group presented a greater incidence of apoptosis and cellular debris (*P* < 0.05) (Fig. 1E, F). Moreover, increased levels of collagenase residue were detected in the L-SVF extraction process (*P* < 0.05) (Fig. 1G).

### C-SVF effectively improved skin wound closure

**Fig. 2:**
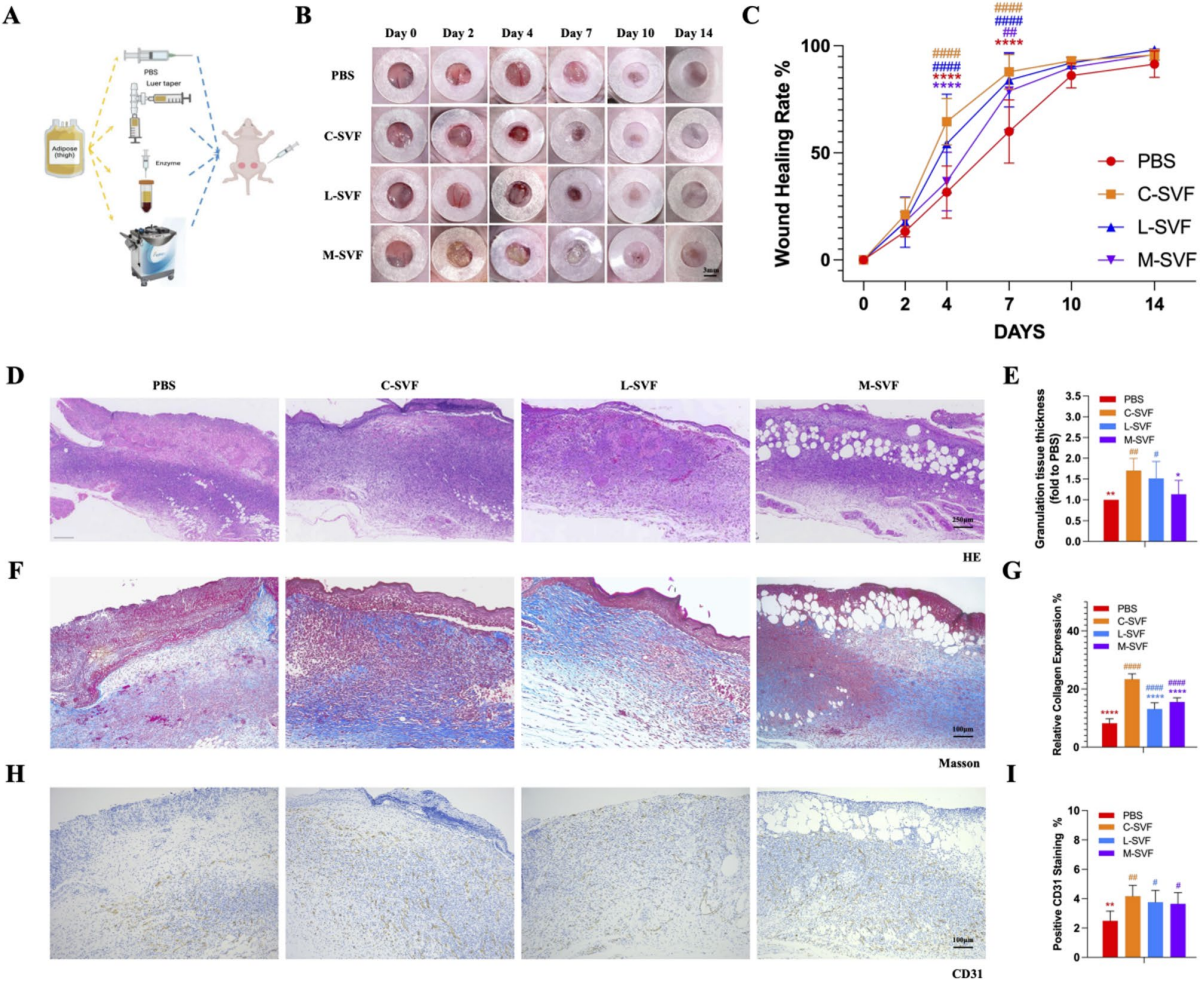
Effects of different SVFs on wound healing in nude mice. A Schematic overview of the experimental flowchart for animal studies involving SVFs procured via diverse methods. B Representative images of full skin wounds in different treatment groups. Scale bar = 3 mm. C Quantitative analysis of the wound healing rate(*n* = 6). D Representative images of H&E-stained wound tissue sections on day 7 following surgery from the PBS, C-SVF, L-SVF and M-SVF groups; scale bar = 250 μm E Quantification of granulation tissue thickness in the wounds on day 7 following surgery (*n* = 6). F Masson’s trichrome staining of the wound tissue sections on day 7 following surgery from the PBS, C-SVF, L-SVF and M-SVF groups; scale bar = 100 μm. G Quantification of collagen volume fractions in the wounds on day 7 following surgery (*n* = 6). H CD31 immunostaining results of the wound tissue sections on day 7 following surgery from the PBS, C-SVF, L-SVF and M-SVF groups; scale bar = 100 μm. I The percentage of CD31-positive cells in the wounds on day 7 following surgery was quantified(*n* = 6). (* represents statistical significance compared with C-SVF; # represents statistical significance compared with PBS. ∗ *P* < 0.05, ∗∗ *P* < 0.01, ∗∗∗ *P* < 0.001, ∗∗∗∗ *P* < 0.0001, two-way ANOVA followed by Tukey’s multiple comparison test)

To compare the regenerative capacity of SVF cells obtained via different methods, we used a full-thickness wound model in nude mice (Fig. 2A). Faster wound healing was observed in both the C-SVF and L-SVF groups (Fig. 2B). By day 7, the wound healing rate for C-SVF reached 87.86%±7.54%. Compared with the C-SVF group, the PBS group showed significantly lower healing (60.01%±13.49%; 95% CI: -41.01 to -14.68, *P* < 0.001), while the L-SVF (83.96%±11.80%; 95% CI: -8.29 to 16.08, *P* = 0.840) and M-SVF groups (77.01%±17.39%; 95% CI: -4.18 to 22.15, *P* = 0.290) showed no significant difference (Fig. 2C). The morphological characteristics of the wounds in each group were observed via H&E and Masson’s trichrome staining. H&E staining on day 7 in each treatment group revealed a significant increase in granulation tissue thickness in the groups treated with C-SVF and L-SVF (compared with the PBS group, the C-SVF and L-SVF groups presented increases of 1.71 ± 0.26-fold and 1.51 ± 0.37-fold, respectively, *P* < 0.05; the M-SVF group presented an increase of 1.13 ± 0.3-fold, *P* > 0.05), indicating more effective re-epithelialization and a well-structured epidermis compared with the other groups. No significant differences were observed between these two groups. In stark contrast, the wound tissue of the M-SVF group contained many mature adipocytes ectopically deposited between the epidermis and dermis (Fig. 2D, E). Furthermore, Masson’s trichrome staining demonstrated increased collagen deposition in the C-SVF-treated group. Compared with the PBS group (8.22 ± 1.40%), the C-SVF, L-SVF and M-SVF groups presented increased collagen deposition (23.38 ± 1.71%, 95% CI: -17.85 to -12.46; 13.14 ± 1.96%, 95% CI: -7.62 to -2.22; and 15.55 ± 1.32%, 95% CI: -10.03 to -4.63, respectively; all *P* < 0.0001)(Fig. 2F, G). Next, we used CD31 for immunohistochemical staining to assess angiogenesis in each group. The results revealed that the C-SVF group had the highest vascular density (4.17 ± 0.68%), which surpassed those of the M-SVF group and L-SVF group (3.65 ± 0.69% and 3.76 ± 0.74%, respectively, *P* > 0.05) (Fig. 2H, I).

### Enrichment of functional cells within the C-SVF

**Fig. 3:**
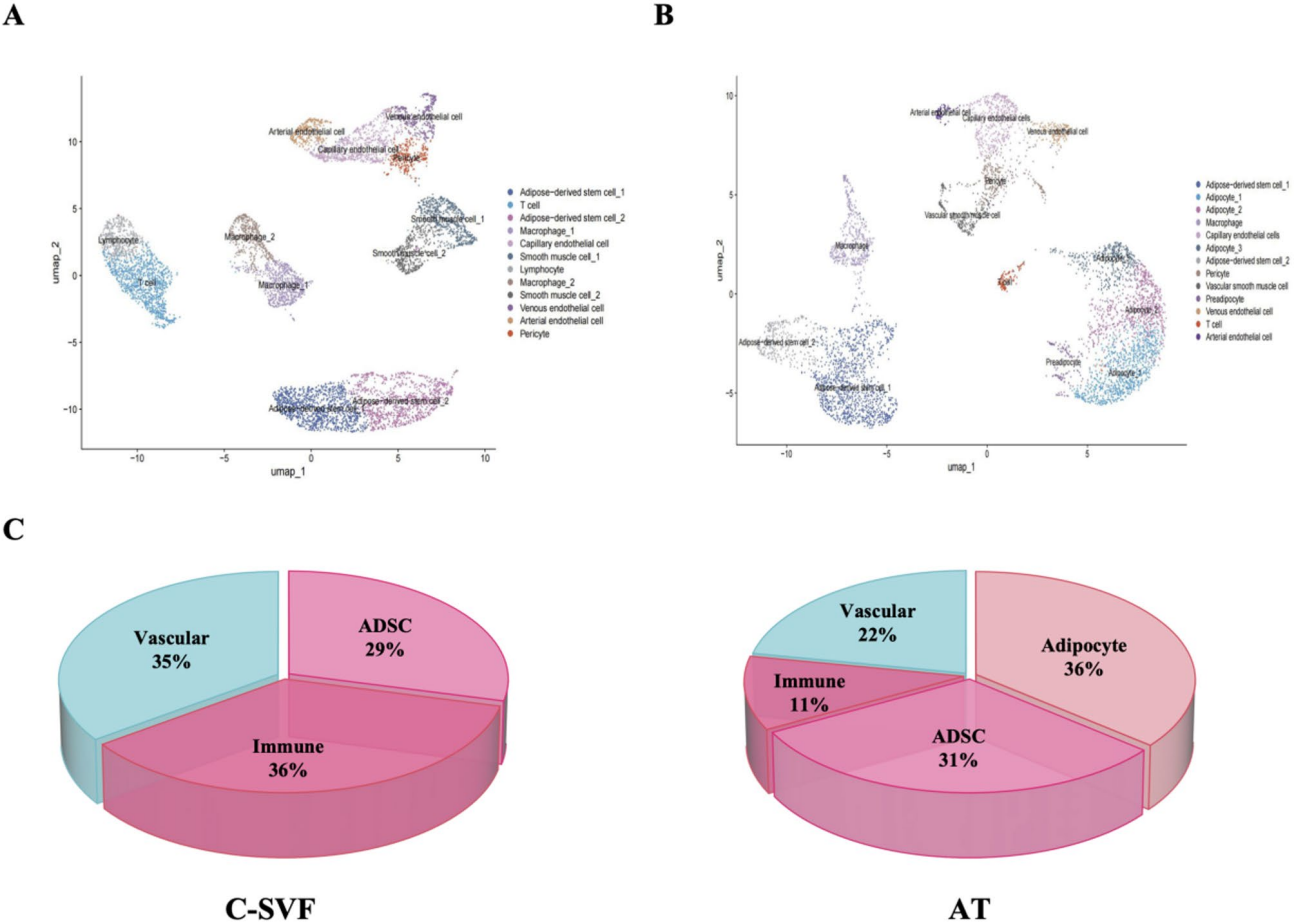
Unsupervised clustering in C-SVF and adipose tissue. (A) For 7,691 cells sampled from whole adipose tissue, unsupervised clustering was carried out, with 11 disparate cell clusters shown on the UMAP plot. (B) For 5,657 cells sampled from whole adipose tissue, unsupervised clustering was carried out, with 12 disparate cell clusters shown on the UMAP plot. (C) A schematic diagram illustrating the relative proportions of diverse cell types in both adipose tissue and C-SVF

To elucidate the cell subpopulations involved in C-SVF and their potential mechanisms, we conducted single-cell nuclear RNA sequencing analysis on subcutaneous adipose tissue and extracted C-SVF from a healthy 23-year-old female. Meanwhile, given the inherent limitation of single-donor sampling in snRNA-seq, we validated key transcriptional patterns using qPCR on three additional donors. Validation data consistently corroborate the generalizability of findings reported below (Supplementary File). By analyzing the sequencing data, the adipose tissue samples and C-SVF samples were each classified into 13 and 12 clusters, respectively (Fig. 3A, B). The results indicate that the identified cell clusters can be categorized into four major groups: adipocytes, ADSCs, vascular cells, and immune cells, which aligns with the findings of Lucas et al. [15]. The large size and relatively fragile nature of mature adipocytes are considered to be the cause of adverse consequences such as oil swelling and inflammation after fat transplantation [16].

Compared with adipose tissue samples, C-SVF samples contain significantly fewer adipocytes. These findings suggest that C-SVF extraction can effectively disrupt and eliminate mature adipocytes, thereby enriching stem cells. Moreover, immune cells and vascular cells were more abundant in the C-SVF.

#### Characterization of ADSCs in C-SVF

**Fig. 4:**
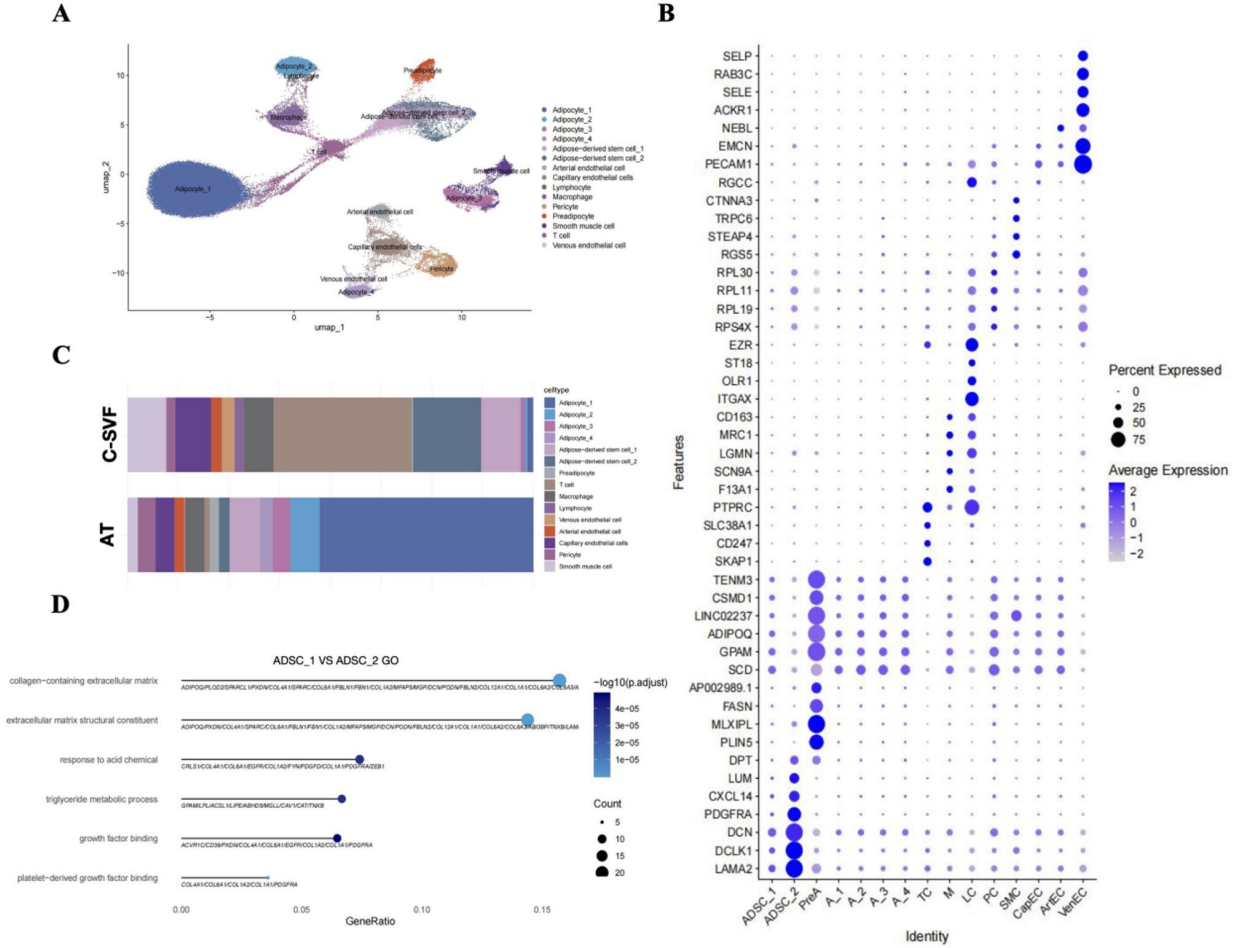
Cell clustering and gene profiling in C-SVF and adipose tissue. A The sample of 24,982 cells underwent unsupervised clustering, and 15 heterogeneous cell clusters were depicted on the UMAP plot. B Dot plot presenting the recognized cell markers and the top 5 differentially expressed genes for each cell population under study. C A stacked bar chart delineating the percentages of assorted cell subtypes within subcutaneous white adipose tissue. D Lollipop plot showing the GO enrichment analysis of upregulated genes in ADSC_2 versus ADSC_1 within the C-SVF sample

To enhance the comparability of these two datasets, we integrated them and conducted quality filtering prior to analysis. Unsupervised clustering of the gene expression profiles revealed 15 distinct cell types (Fig. 4A). The cell clusters were annotated on the basis of the DEGs and established marker genes (Fig. 4B and Additional file 2). By statistically comparing the proportions of the nuclei of major cell types, we found that C-SVF contains two main subpopulations of ADSCs, with a greater percentage of Type 2 ADSCs (Fig. 4C). GO analysis revealed that pathways related to collagen and growth factor synthesis were upregulated in type 2 adipose-derived stem cells compared with type 1 adipose-derived stem cells (Fig. 4D). These findings indicate that the remarkable therapeutic effects of C-SVF on ECM deposition may be associated with the abundant content of type 2 ADSCs.

**Fig. 5:**
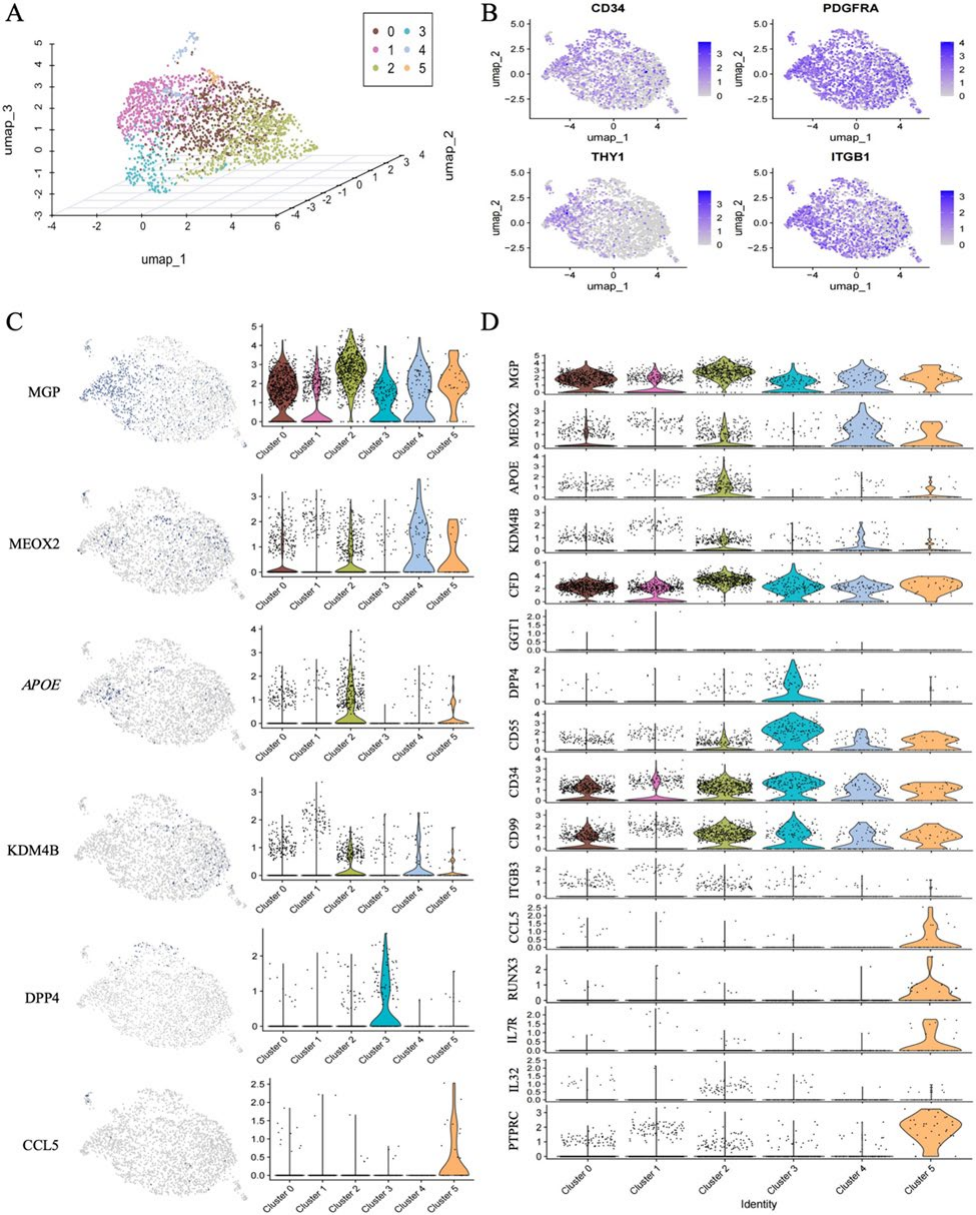
Cell clustering and gene pattern visualization in C-SVF-derived ADSCs. A Unsupervised clustering was conducted on 2,255 adipose-derived stem cells procured from sample C-SVF. A three-dimensional UMAP map was then generated, revealing six distinct cell clusters. B) Investigation of the gene expression patterns of stem cell-specific marker genes derived from adipose tissue within the ADSCs of C-SVF. C) Utilizing individual gene UMAP and violin plots to visualize both the expression levels and distributions of representative marker genes. Notably, the y-axis represents the normalized read count, presented on a logarithmic scale. D) Violin plots were generated to depict the relative log2 expression levels of selected genes across the six groups

To classify different subpopulations of ADSCs more precisely, we reclustered the cells at a higher resolution, dividing the ADSCs into 6 subtypes (Fig. 5A, Additional file 2), all of which significantly expressed the identified ADSC markers Pdgfrα (validated 30-fold higher in C-SVF, *p* = 0.03), CD34, Itgb1 (CD29), and Thy1 (Cd90) [17–20] (Fig. 5B). Among them, clusters 0 and 4 expressed marker genes for adipogenesis-regulating cells (Aregs) as defined by Schwalie et al. [21]. The tendency of Aregs to form adipocytes is significantly decreased and can negatively regulate the adipogenic capacity of other ADSCs through paracrine signaling mechanisms, such as Rtp3, Spink2, Fgf12, and Vit [21, 22].

Cluster 1 exhibited relatively high expression of KDM4B, a gene known to be linked with the transcriptional activation of various metabolic genes, including PGC-1. Mice lacking KDM4B display compromised adrenergic responses and albinism in their brown adipose tissue [23]. Consequently, Cluster 1 may indicate potential beige adipocyte precursors within white adipose tissue. In Cluster 2, the APOE gene is significantly overexpressed, and its functions encompass multidimensional regulatory mechanisms. On the one hand, it facilitates adipocyte differentiation by activating the PPARγ pathway [24]. On the other hand, it neutralizes proinflammatory factors (such as TNF-α and IL-6) to inhibit the activation of the NF-κB pathway while also promoting the polarization of M2 macrophages, thus forming an immunosuppressive microenvironment [25, 26]. These functions suggest that Cluster 2 genes may be involved in maintaining metabolic homeostasis and tissue repair by regulating lipid metabolism and the immune microenvironment. Cluster 3 cells display high expression of Dpp4 and CD55. Research indicates that the expression levels of general stem cell markers (such as CD34 and CD73), genes linked to cancer stem cells (CD99 and ITGB3), and an embryonic stem cell marker (GGT1) are elevated within this subset [27, 28]. These findings suggest that this cell subset may have enhanced self-renewal and proliferation capabilities. Further studies have demonstrated that this subset has superior therapeutic effects in promoting wound healing and regeneration [29]. Additionally, Cluster 3 shows a certain level of CD24 expression, and CD24 + cells have been previously confirmed to be adipogenic progenitor cells capable of fat formation in vivo [20]. Therefore, this subset may also play a significant role in the process of adipocyte differentiation.

In cluster 5, transcriptomic analysis revealed significantly elevated expression levels of RUNX3 and CCL5 (validated 8.6-fold higher in C-SVF, *P* = 0.04). Functional validation studies have demonstrated that ADSCs exert proangiogenic effects on human umbilical vein endothelial cells (HUVECs) through RUNX3-mediated signaling pathways, promoting endothelial cell proliferation, migration, and tube formation [30]. Subsequent in vivo investigations further identified CCL5 as a critical effector molecule in ADSC-driven angiogenesis during cutaneous wound healing [31]. Additionallly, cluster 5 cells expressed high levels of inflammatory markers (such as *Ccl5*,* IL7R*,* Ptptc*, and *IL32*), indicating that cluster 5 cells might orchestrate wound healing through bidirectional crosstalk with infiltrating immune cells [32]. Collectively, these results suggest that cluster 5 may have potential application value in modulating inflammation resolution and angiogenesis (Fig. 5C, D).

#### Characterization of immune cells in the C-SVF

On the basis of the above analysis, we determined that specific ADSC subsets dynamically interact with macrophages during wound repair. Notably, T lymphocytes were significantly enriched in the C-SVF fraction, constituting the dominant cell population (Fig. 4C). To characterize these interactions in detail, we performed a comprehensive analysis of immune cell subsets within the C-SVF, categorizing them into two major lineages: (i) lymphoid cells dominated by T cells and (ii) myeloid cells comprising mainly M2-like macrophages [33]. Sngle-cell profiling of these populations enabled the construction of a high-resolution atlas defining the transcriptional diversity and functional heterogeneity of distinct immune cell subsets.

**Fig. 6:**
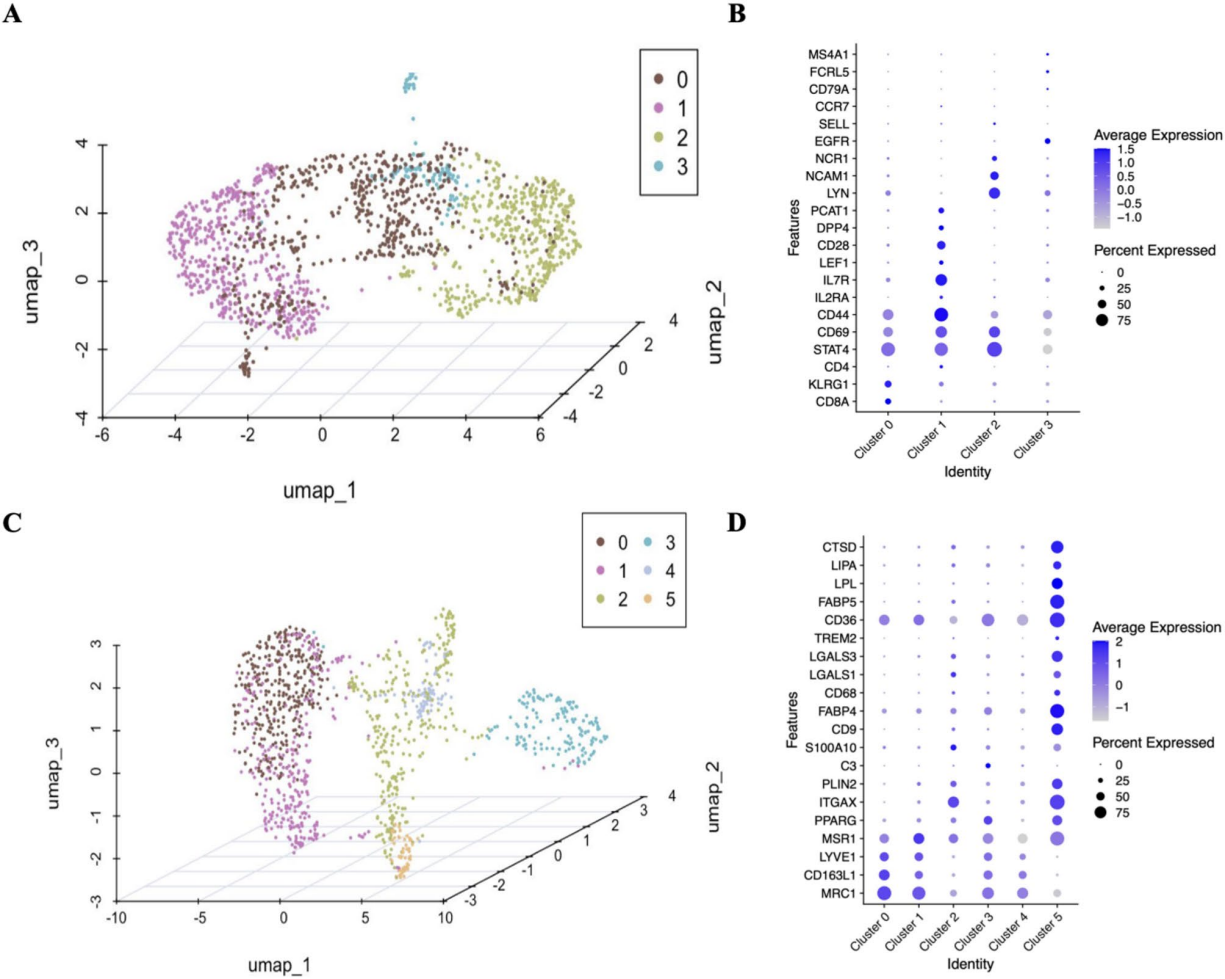
Clustering and marker analysis of immune cells in the C-SVF. Unsupervised clustering analysis was performed on 1,575 lymphocytes sampled from C-SVF, and a three-dimensional UMAP map was constructed, which revealed four discrete cell clusters. A dot plot was generated to display selected cell markers corresponding to each lymphocyte subpopulation. Similarly, unsupervised clustering was carried out on 1,191 macrophages obtained from sample C-SVF, yielding a three-dimensional UMAP map that revealed six distinct cell clusters. A dot plot was subsequently devised to show selected cell markers specific to each macrophage subpopulation

In the major lymphocyte group, four distinct clusters (clusters 0–3 in descending abundance, Fig. 6A, Additional file 2) were identified. Cluster 0 presented relatively high expression of CD8A. Research suggests that the CD8α protein encoded by CD8A may participate in ADSC–immune cell crosstalk and regulate local immune responses [34]. Cluster 1 highly expresses CD28 and PCAT-1 and contains genes related to regulatory T-cell (Treg) subtypes [35]. In vitro, ADSCs can promote paracrine-mediated anti-inflammatory events by decreasing CD28-T cells and increasing FoxP3 + Tregs [36]. Elevated PCAT-1 levels are associated with reduced immune cell infiltration [37]. Cluster 2 had relatively high CTBP2 expression. Notably, LW et al. reported that miR-342-3p from human ADSCs can inhibit CtBP2 to activate adipogenic factor and marker expression [38]. Cluster 3 expressed TRM and B-cell marker genes [35] and highly expressed EGFR (validated 35-fold higher in C-SVF, *P* = 0.01). A previous study [39] indicated that human ADSCs can activate skin stem cells via the EGFR/MEK/ERK pathway to promote wound healing (Fig. 6B).

The myeloid group was classified into six clusters (clusters 0–5, Fig. 6C, Additional file 2). Clusters 0 and 1 highly expressed LYVE1, which is potentially involved in tissue support and angiogenesis [40, 41]. Notably, Cluster 0 exhibited marked upregulation of CD163L1 (validated 95-fold higher in C-SVF, *P* = 0.003), an endocytic receptor whose expression is induced during monocyte-to-macrophage differentiation under M-CSF stimulation but suppressed by proinflammatory mediators such as TNF-α [42]. Cluster 2 expressed the marker genes of metabolically regulated macrophages [15], which are important for regulating inflammatory mediator and lipid metabolism balance [43, 44]. Additionally, cluster 2 and 3 relatively highly expressed genes related to cell‒matrix interactions and ECM remodeling, such as *C3* and *S100A10* [45, 46]. Cluster 5 also expressed genes related to lipid-associated macrophages (LAMs) (such as *CD9*,* ABP4*,* CD68*, and *N1*) [47–49]. LAMs express many genes related to immunosuppression (e.g., Lgals1 and Lgals3), suggesting that they might be involved in regulating inflammatory responses related to cell death and lipid accumulation [50]. Burl et al. [51, 52] identified a macrophage cluster (cluster 5 highly expressed relevant genes) and suggested that these cells might provide growth factors for adipose stem cells (Fig. 6D).

On the basis of the above analysis, we posit that within the C-SVF fraction, ADSC subsets and diverse immune cell populations likely form a bidirectional regulatory network via paracrine signaling. ADSCs suppress excessive inflammation and remodel the immune microenvironment through the secretion of bioactive substances, whereas immune cells reciprocally promote ADSC proliferation, differentiation, and angiogenic gene expression via cytokine release. This dynamic interplay results in a multidimensional regulatory network for wound healing. The complete qPCR validation data can be found in theSupplementary File.

## Discussions

The SVF is a rich source of ADSCs and other therapeutically potent cell types, including vascular endothelial cells and tissue macrophages, which are crucial for wound healing and tissue regeneration. Currently, enzymatic digestion is the predominant method for SVF isolation. This well-established technique yields relatively high cell numbers and viability, and is progressively evolving toward closed, automated systems [53]. Consequently, various semi-automatic and fully automatic SVF isolation devices have been introduced to the market in recent years. Preclinical and clinical studies demonstrate that SVF cells promote angiogenesis [12, 54], reduce inflammation [11], and enhance other parameters related to the healing mechanism [55, 56].

Our findings demonstrate that the production efficiency of C-SVF is comparable to that of manual enzymatic digestion. However, the Celution system offers distinct advantages, including reduced procedure time, simplified workflow, lower risk of exogenous contamination, decreased operator dependency, and enhanced reproducibility. Furthermore, commercial extraction kits typically yield lower residual collagenase levels. While regulatory perspectives on enzyme use vary globally, a common objective is to minimize exogenous additives. These results suggest that C-SVF represents a promising alternative stem cell source. Although M-SVF may not be optimal for promoting tissue regeneration per se, its role in volumetric defect reconstruction remains indispensable. Consequently, a future strategy could involve combining C-SVF with conventional M-SVF. This approach may augment the functional cell content within the M-SVF graft while preserving its critical volumetric capacity, potentially leading to superior therapeutic outcomes.

Furthermore, we present the first snRNA-seq analysis of SVF isolated using commercial closed systems. By integrating and comparing these data with sequencing results from intact adipose tissue, we identified ADSCs as a predominant cellular component within C-SVF, suggesting their significant functional potential. Further subclustering analysis revealed six functionally heterogeneous ADSC subsets, each exhibiting distinct molecular profiles and clinical relevance. For instance, high expression of KDM4B in Cluster 1 correlated significantly with the activation of PGC-1α-mediated metabolic genes. This epigenetic mechanism drives the browning of white adipose tissue by enhancing mitochondrial biogenesis. Our prior work demonstrated that the emergence of beige adipocytes within wound tissue promotes repair [57]. Subsequent secretion of brown adipokines further accelerates metabolic reprogramming, collagen deposition, and angiogenesis in the wound microenvironment [58]. Cluster 2 exhibited dual APOE-dependent regulation of both the immune microenvironment and lipid metabolism. Functional validation confirmed that APOE-overexpressing ADSCs significantly enhanced healing in diabetic ulcers, establishing APOE as a metabolic‒immunomodulatory hub [59, 60]. Collectively, these findings demonstrate that distinct ADSC subpopulations facilitate wound repair through a coordinated multi-mechanism network involving metabolic reprogramming, immune modulation, and angiogenic differentiation.

Single-cell analysis suggested that ADSCs establish a multidimensional regulatory network with immune cells (T cells and macrophages) primarily through paracrine signaling. Notably, T cells within wound tissue exhibit marked heterogeneity (Clusters 0–3). ADSCs appear to promote T regulatory cell (Treg; Cluster 1) proliferation while suppressing CD8 + T-cell activity [61]. Analysis of macrophage subsets revealed that C-SVF is enriched in M2 anti-inflammatory macrophages. These cells attenuate excessive inflammation via anti-inflammatory cytokine secretion (e.g., IL-10, TGF-β) [62], stimulate angiogenesis through vascular endothelial growth factor release, and facilitate tissue remodeling by promoting collagen matrix synthesis [63, 64]. Furthermore, Cluster 5 macrophages, characterized by high expression of immunosuppressive factors like Lgals3, engage in a symbiotic relationship with ADSCs to foster proliferation, establishing an immunosuppressive-stem cell activation niche. Collectively, these findings provide a novel perspective on the immunometabolic regulation underlying wound repair. Future research should integrate spatial transcriptomics and single-cell epigenomics to elucidate the dynamic inter-subset communication networks and validate their translational potential as therapeutic targets.

This study has several limitations. We faced limited donor availability and lacked long-term functional outcome data. First, in the meta-analysis by Massier et al. [15], the correlation between snRNA-seq data and the transcriptional profiles of isolated adipocytes was weak. Our snRNA-seq data detected only low-expression adipocyte subtype marker genes (e.g., Lep, Saa1, Rbp4) [65]. Consequently, determining adipocyte subtypes requires integrated analysis using complementary technical platforms, such as spatial transcriptomics. Second, our sequencing results are derived from a single sample, which restricts the generalizability of the findings. Nevertheless, we validated key observations using qPCR on three independent donor pairs. Finally, future studies are needed to investigate the long-term effects of these technologies and cell populations. A deeper understanding of the underlying mechanisms could enable targeted improvements in the cell extraction process, thereby enhancing therapeutic efficacy and expanding applications in regenerative medicine.

## Conclusions

Our research demonstrates that C-SVF effectively promotes wound healing through potential mechanisms suggested by snRNA-seq, including enrichment of specific regenerative cell subpopulations, enhanced ECM remodeling, and augmented immunomodulatory functions.

## Data Availability

The single-nucleus RNA sequencing datasets generated and analyzed during the current study are available in public repositories to ensure full accessibility and reproducibility. The raw sequencing data have been deposited in the NCBI Sequence Read Archive (SRA) under BioProject accession number PRJNA1332371. The processed gene expression matrices, analyzed data, and complete metadata have been deposited in the NCBI Gene Expression Omnibus (GEO) under accession number GSE308930. These datasets will be publicly released upon article publication. All other experimental materials are available from the corresponding author upon reasonable request.

## Abbreviations

ADSCs: Adipose-derived stem cells
SVF: Stromal vascular fraction
ECM: Extracellular matrix
ADRCs: Adipose-derived regenerative cells
snRNA-seq: Single-nucleus RNA sequencing
L- SVF: SVF Prepared by laboratory enzymatic digestion
rpm: Revolutions per minute
PBS: Phosphate buffered saline
M- SVF: SVF generated by mechanical emulsification
C-SVF: SVF generated by commercial cell separation systems
DMEM: Dulbecco’s modified eagle medium
OD: Optical density
H&E: Hematoxylin and eosin
EDTA: Ethylenediaminetetraacetic acid
HRP: Horseradish peroxidase
qRT-PCR: Quantitative reverse transcription polymerase chain reaction
WAT: White adipose tissue
GEMs: Gel bead in emulsion microdroplets
UMI: Unique molecular identifiers
UMAP: Uniform manifold approximation and projection
PCA: Principal component analysis
DEG: Differentially expressed gene
GO: Gene ontology
ECs: Endothelial cells
EPCs: Endothelial precursor cells
Aregs: Adipogenesis-regulating cells
HUVECs: Human umbilical vein endothelial cells
Tregs: Regulatory T cells
LAM: Lipid associated macrophage
CE: Onformité européenne
